# An information value based analysis of physical and climatic factors affecting dengue fever and dengue haemorrhagic fever incidence

**DOI:** 10.1186/1476-072X-4-13

**Published:** 2005-06-08

**Authors:** Kanchana Nakhapakorn, Nitin Kumar Tripathi

**Affiliations:** 1 Remote Sensing and GIS field of study, Asian Institute of Technology, Pathumthani, Thailand

## Abstract

**Background:**

Vector-borne diseases are the most dreaded worldwide health problems. Although many campaigns against it have been conducted, Dengue Fever (DF) and Dengue Haemorrhagic Fever (DHF) are still the major health problems of Thailand. The reported number of dengue incidences in 1998 for the Thailand was 129,954, of which Sukhothai province alone reported alarming number of 682. It was the second largest epidemic outbreak of dengue after 1987. Government arranges the remedial facilities as and when dengue is reported. But, the best way to control is to prevent it from happening. This will be possible only when knowledge about the relationship of DF/DHF with climatic and physio-environmental agents is discovered. This paper explores empirical relationship of climatic factors rainfall, temperature and humidity with the DF/DHF incidences using multivariate regression analysis. Also, a GIS based methodology is proposed in this paper to explore the influence of physio-environmental factors on dengue incidences. Remotely sensed data provided important data about physical environment and have been used for many vector borne diseases. Information Values (IV) method was utilised to derive influence of various factors in the quantitative terms. Researchers have not applied this type of analysis for dengue earlier. Sukhothai province was selected for the case study as it had high number of dengue cases in 1998 and also due to its diverse physical setting with variety of land use/land cover types.

**Results:**

Preliminary results demonstrated that physical factors derived from remotely sensed data could indicate variation in physical risk factors affecting DF/DHF. A composite analysis of these three factors with dengue incidences was carried out using multivariate regression analysis. Three empirical models ER-1, ER-2 and ER-3 were evaluated. It was found that these three factors have significant relation with DF/DHF incidences and can be related to the forecast expected number of dengue cases. The results have shown significantly high coefficient of determination if applied only for the rainy season using empirical relation-2 (ER-2). These results have shown further improvement once a concept of time lag of one month was applied using the ER-3 empirical relation. ER-3 model is most suitable for the Sukhothai province in predicting possible dengue incidence with 0.81 coefficient of determination. The spatial statistical relationship of various land use/land cover classes with dengue-affected areas was quantified in the form of information value received from GIS analysis. The highest information value was obtained for the Built-up area. This indicated that Built-up area has the maximum influence on the incidence of dengue. The other classes showing negative values indicate lesser influence on dengue epidemics. Agricultural areas have yielded moderate risk areas based on their medium high information values. Water bodies have shown significant information value for DF/ DHF only in one district. Interestingly, forest had shown no influence on DF/DHF.

**Conclusion:**

This paper explores the potential of remotely sensed data and GIS technology to analyze the spatial factors affecting DF/DHF epidemic. Three empirical models were evaluated. It was found that Empirical Relatrion-3 (ER-3) has yielded very high coefficient of determination to forecast the number of DF/DHF incidence. An analysis of physio-environmental factors such as land use/ land cover types with dengue incidence was carried out. Influence of these factors was obtained in quantitative terms using Information Value method in the GIS environment. It was found that built-up areas have highest influence and constitute the highest risk zones. Forest areas have no influence on DF/DHF epidemic. Agricultural areas have moderate risk in DF/DHF incidences. Finally the dengue risk map of the Sukhothai province was developed using Information Value method. Dengue risk map can be used by the Public Health Department as a base map for applying preventive measures to control the dengue outbreak. Public Health Department can initiate their effort once the ER-3 predicts a possibility of significant high dengue incidence. This will help in focussing the preventive measures being applied on priority in very high and high-risk zones and help in saving time and money.

## Background

Vector borne diseases are the most common worldwide health hazard and represent a constant and serious risk to a large part of the world's population. Among these, dengue fever especially is sweeping the world in majority of the tropical and arid zones. It is transmitted to the man by the mosquito of the genus *Aedes *and exists in two forms: the Dengue Fever (DF) or classic dengue and the Dengue Haemorrhagic Fever (DHF), which may evolve into Dengue Shock Syndrome (DSS)[1]. Dengue infection occurs due to the bite of the mosquito *Aedes aegypti*, that is infected with one of the four dengue virus serotypes [2]. The infection, earlier restricted to urban/semi-urban centres, can now be seen in rural areas as well [3]. Land use/ land cover types and climate play significant role in dengue cases as reported by several researchers [3]. Remotely sensed data can be used to identify, monitor and evaluate environmental factors between vector and environment relationships. Recently, Geographic Information Systems (GIS) and Remotely Sensed data are being used to evaluate and model the relationships between climatic and environmental factors with the incidences of viral diseases. Spatial analysis involves the use of Geographic Information Systems (GIS) for health that has been reviewed by several authors [4-9]. Both spatial and temporal changes in environmental condition may be important determinants of vector-borne disease transmission. Remote sensing data can be used to provide information on the spatial distribution of the vector-borne diseases and the physical environment [10-12]. It is mentioned by a researcher that remote sensing and geodesy have the potential to revolutionize the discipline of epidemiology and its application in human health [13].

Remote sensing and other types of data were used in GIS to identify villages at high risk for malaria transmission in the southern area of Chiapas, Mexico [12]. In Nigeria, a temporal analysis of Landsat Thematic Mapper (TM) satellite data was carried out to test the significance of the guinea worm eradication program based on changes in agricultural production [11]. And, it was also employed to predict and map the location of some of the major diseases affecting human health as well [10,14]. Land use/ land cover types are the critical variables in epidemiology and can be characterized by remote sensing [15]. At the same time, it was observed that use of remote sensing and GIS in health sector is quite limited in Thailand.

Dengue Fever (DF) and Dengue Haemorrhagic Fever (DHF) has become a major international public health concern. Many countries/areas in Asia have been experiencing unusually high levels of dengue/dengue haemorrhagic fever activity in 1998 [16]. The reported number of dengue incidences in 1998 for the Thailand was 129,954, which was the second largest epidemic outbreak of dengue after 1987. In 1998, Sukhothai province, Thailand alone reported alarming number of 682 dengue cases.

A GIS based methodology is proposed in this paper to explore the influence of physio-environmental and climatic factors on dengue incidences. Information Values (IV) method was utilised to derive influence of various physio-environmental factors in the quantitative terms. Researchers have not applied this method of analysis for dengue earlier. Sukhothai province was selected for the case study as it had high number of dengue cases in 1998 and also due to its' diverse physical setting with variety of land use and land cover types.

## Data and methods

### Study area and data used

**Figure 1:**
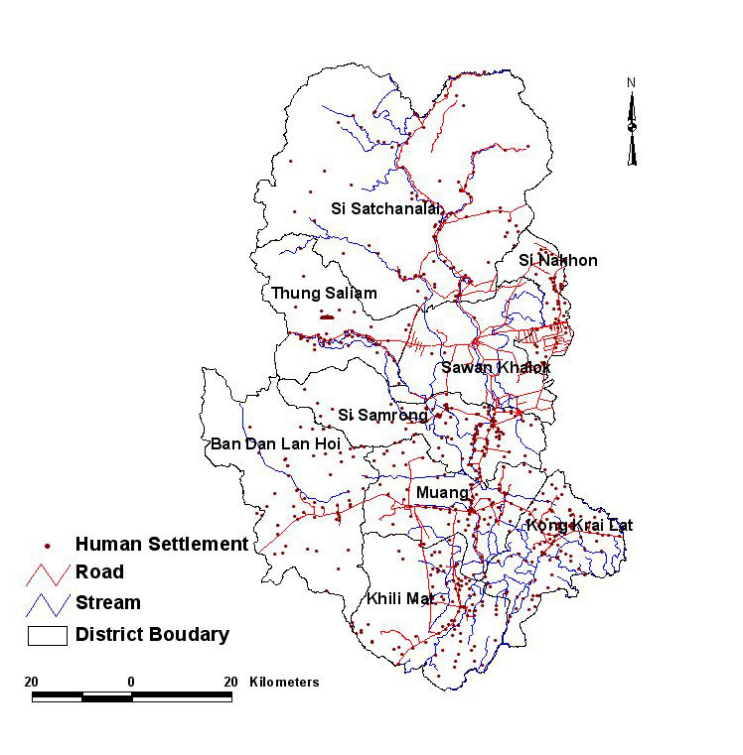
Location map of study area, Sukhothai, Thailand.

Sukhothai province, located in the northern Thailand, was selected as the study area (Figure 1). This province consists of 9 Districts: Muang Sukhothai (D1), Ban Dan Lan Hoi (D2), Khiri Mat (D3), Kong Krailat (D4), Sawankhalok (D5), Si Nakhon (D6), Si Samrong (D7), Si Satchanalai (D8) and Thung Saliam (D9). GIS database was developed for each of the districts. People are predominantly involved in the agriculture such as sugarcane, cassava, and corn. The province has a population of about 521,219. Climate of this area is subtropical with extreme high temperatures rising to 42°C in April and dipping low up to 13.2°C in December. In 1998, the average annual temperature was 28°C, which was higher than the 30 Year average normal temperatures of 26°C. Rainy season in Thailand normally occurs from May to September. The daily maximum and annual rainfall are 62.5 mm and 917.7 mm respectively. The average relative humidity is 94.8 percent.

### Medical data

Medical data were collected from the Provincial Health Office, Thailand, which collects monthly district level data. In 1998, 682 DF/DHF cases were reported in Sukhothai Province. Morbidity rate was observed at 131 per 100,000 people. The DHF incidences were recorded at the village level. Highest numbers of dengue incidence were recorded in districts Muang, Si Satchanalai and Sawan Khalok (Table 1). It was found that highest number of cases occurred during March and August. This indicated the seasonal dependence in occurrence of DF/DHF cases, which generally starts just before the rainy season and continues till the end of rainy season.

**Table 1:** Monthly distribution of DF/DHF cases in Sukhothai province in 1998

District Name	Jan	Feb	Mar	Apr	May	Jun	Jul	Aug	Sep	Oct	Nov	Dec	Total
Muang Sukhothai Thani	18	31	40	7	3	1	3	15	16	5	2	0	141
Kong Krai Lat	3	5	6	2	2	3	6	4	4	0	0	0	35
Khili Mat	15	8	21	12	6	14	11	8	5	2	0	0	102
Thung Saliam	5	1	4	4	3	1	3	14	4	3	4	6	52
Ban Dan Lan Hoi	6	5	3	6	6	9	8	9	3	4	0	0	59
Si Satchanalai	5	4	7	9	23	14	20	22	6	2	7	4	123
Si Samrong	5	3	7	3	4	9	9	4	7	0	0	0	51
Sawan Khalok	12	4	15	12	5	2	11	15	7	7	9	7	106
Si Nakhon	1	1	3	3	0	0	0	4	1	0	0	0	13

### Factors influencing dengue

Major factors considered for analysis of the occurrence of DF/DHF cases were rainfall, temperature, humidity, and land use/land cover types. DF/DHF outbreaks in Sukhothai, Thailand occurred in 1997, 1998 and 2001. It was noticed that the dengue outbreak coincided with El Nino years. El Nino events in Thailand are normally associated with high temperature and low precipitation. The monthly rainfall, temperature and relative humidity data were collected from the Department of Meteorology, Ministry of Information and Communication Technology, Thailand. Thailand experiences rains from May to September and remaining part of the year remains mostly dry. In addition to the rainfall, temperature, and humidity also influence dengue transmission [17]. Due to high humidity during rainy season mosquito survival is longer and growth is facilitated [18]. The average temperatures in Sukhothai province were between 22°C and 33°C in 5 years (1997–2001). Higher than 20°C is the favourable temperature for *Aedes aegypti *mosquitoes [2]. The average relative humidity observed in 5 years (1997–2001) was 95.6 percent. The 30 years (1969–2000) average monthly rainfall was 1226.5 mm in Northern Regions of Thailand. As the season in Thailand can be divided in broader context into two types: rainy and non-rainy, the effect of these three factors on dengue was analysed for these two parts of the year.

### Physio-environmental factors

**Figure 2:**
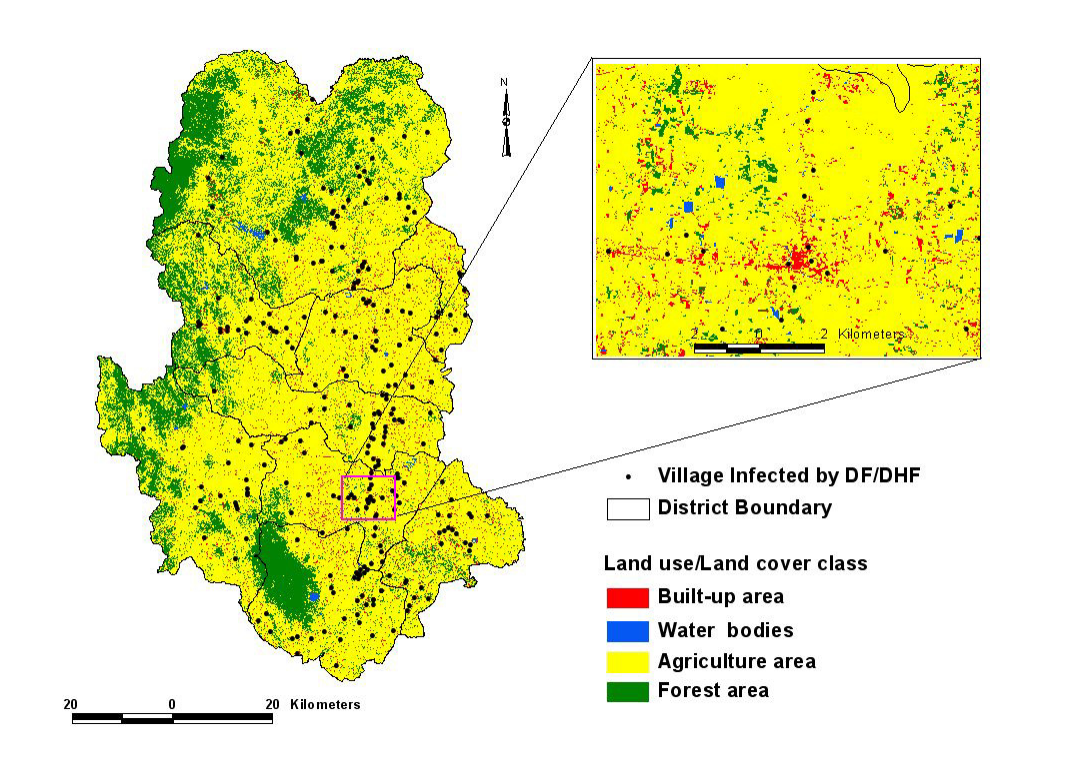
Land use/land cover map showing DF/DHF affected settlement in Sukhothai, Thailand.

The area of Sukhothai province is 6,694.54 km^2^. Digital remote sensing data from Landsat (Thematic Mapper) were employed to produce the land cover type map using the Maximum Likelihood Classifier (MLC). Various output classes generated were subsequently verified based on the field observations. Maximum Likelihood Classifier offered 86 percent classification accuracy. The satellite data were geo-referenced using the Royal Thai Survey Department (RTSD) base map. The GIS analysis revealed the land use areas as: agricultural (74.7%), forest (21%), water bodies (0.3%) and Built-up (4.0%) for the year 1998. Resultant map from the classification is shown in the figure 2.

### Methodology

Dependence of the climatic variables was evaluated using multiple regression analysis. In addition, as suggested by many researchers, physio-environmental factors also affect dengue incidence. Information value approach was utilized to explore which physical and environmental factors are more crucial in dengue incidences.

### Relationship of dengue incidence with climatic factors using multiple regression analysis

The general purpose of multiple regressions is to learn more about the relationship between several independent or predictor variables and a dependent or criterion variable. The general computational problem that needs to be solved in multiple regression analysis is to fit a straight line to a number of points. In the multivariate case, when there is more than one independent variable, the regression line cannot be visualised in the two-dimensional space, but can be computed just as easily. It is possible to construct a linear equation containing all variables. In general multiple regression procedures will estimate a linear equation of the form:

Y = b_0_+b_1_X_1_+b_2_X_2_+...+b_k_X_k_

Where k is the number of predictors. Note that in this equation, the regression coefficients (or b_0_, b_1_, b_2_...b_k _coefficients) represent the independent contributions of each independent variable to the prediction of the dependent variable [19]. In present study climatic independent variables such as rainfall, temperature and humidity were related to the dengue cases in the Sukhathai province. The application of this method is detailed in following section of Results.

### Information value method

An understanding of spatial relationship of Dengue epidemic with affecting factors is essential before applying any statistical models to find the influence of factors in dengue epidemic. The simple technique to understand the statistical relationship is conditional analysis, which attempts to assess the probabilistic relationship between relevant factors affecting dengue epidemic and environmental factors. The technique is based on Baye's theorem (Bayesian classifier) according to which frequency data can be used to calculate the probabilities that depends upon the knowledge of previous events or dengue epidemic/outbreak [20-22].

The information value equation is expressed as the natural logarithmic ratio of conditional probability of the thematic feature.



Where, I_j _is the predictive information of occurrence of event *D *if feature *A *is present under state *j*

*P{D/Aj} *is the conditional probability of event *D *to occur under the condition of feature *A *and state *j*.

This is the same as the conditional probability of dengue epidemic *(D) *to occur because of spatial feature *(A*) present in one of the thematic(jth) layer.

*P{D} *is the probability that event *D *will happen in the selected area irrespective of any evidence.

Map crossing results in a cross table showing the number of pixels per class occupied by dengue epidemic and total number of pixels in each class. The remaining values, necessary to calculate information value, area obtained from these values, actually can represent in term of information value process (Figure 2), therefore



where,

i-value = information value

ndclass = area with dengue epidemic in a class(i.e. land use/land cover types in the buffer zone)

nclass = area in the class (i.e. land use/cover types by district)

ndmap = total area of dengue epidemic in the map (thematic layer)

nmap = total area in the map

**Figure 3:**
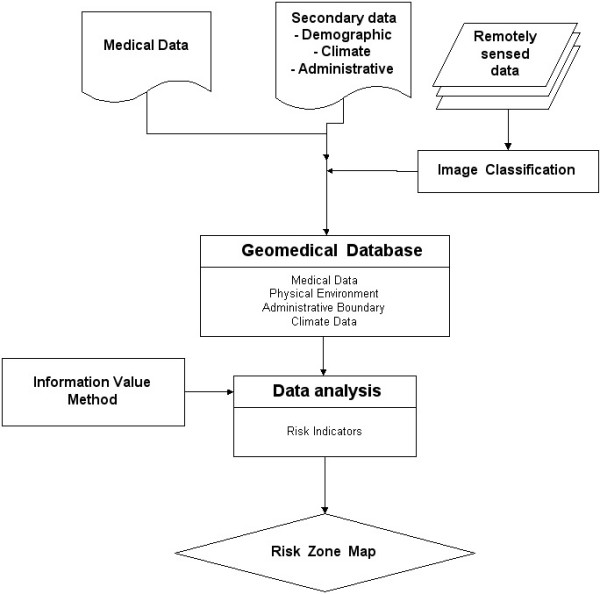
Methodology for mapping DF/DHF risk zones using Information Value approach.

Information value of various parameters can be utilized to interpret the relationship of the parameters and dengue incidences (Figure 3).

### Buffering

Buffering operation was carried out to determine extended neighbourhood of place of occurrence. Results from the human settlement i.e. urban or sub-urban centre in Sukhothai province were obtained from maps obtained from Ministry of Interior, Thailand. Buffers were created to specify distance of the point pattern of disease from urban/ village locations and to identify the geographic environment conditions such as land cover, water bodies, surrounding village affected by DHF. The buffer distance was considered owing to two factors: flight distance covered during the life span and average distance travelled per day by the *Aedes aegypti *mosquito. The average lifespan of female is about 8–15 days and the female mosquito can fly about 30–50 m per day on an average. This indicates that in general female mosquito would move around 240–600 m range in their lifetime [23]. These buffer zones were divided into two groups according to the distance from life span, and average distance travelled per day by the *Aedes aegypti *mosquito. Group 1 was composed of areas within 500 m and Group 2 composed of an area of 1000 m around the dengue incidence. Therefore, the buffer zones of 500 m and 1000 m were created using GIS for this study.

## Results and discussion

Regression analysis was used to explore the relationship between the monthly climatic parameters and the number of incidences of DF/DHF in Sukhothai province.

### Model development

Multiple regression analysis is employed to develop an empirical model to predict the dengue incidences. The independent variables were used to predict changes in the dependent variable in the rainy and non-rainy seasons. This model was verified using the R^2 ^statistics. The variables used in the models are explained in the Table 2.

**Table 2:** Variable in the multiple regression models

Dependent variable	
D_t_	Number of DF/DHF cases based on monthly report from the Sukhothai Provincial Public Health office, Ministry of Public Health
Independent variables	
T	Maximum monthly temperature(°C)
R	Total monthly rainfall (mm./month)
H	Maximum monthly relative humidity(%)
*t*	time in month
*t-1*	one month before *t*

**Figure 4:**
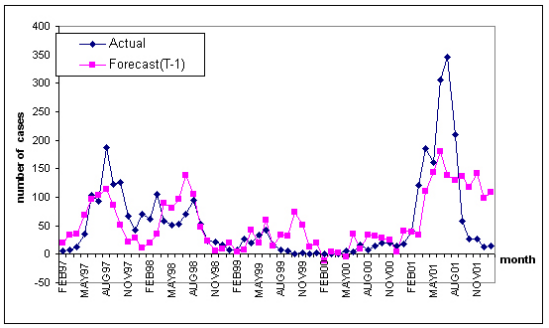
Relationship between actual and forecasted DF/DHF cases in Sukhothai, Thailand.

Number of peoples affected by DF/DHF was used as the dependent variable and the rainfall(R), temperature(T) and relative humidity(H) were considered as the independent variables. Multiple regression analysis was carried out for each of the observations of the occurrence of DF/DHF cases and monthly climatic data of 5 years (1997–2001). The Empirical Relationship-1 (ER-1) between number of DF/DHF cases and the climatic data at time *t *(T_*t*_, R_*t *_and H_*t*_) during 5 years is listed in ER-1. The coefficient of determination (R^2^) was found as 0.43 and validated with climate data as shown in the graph in Figure 4.

ER-1:

D_*t *_= 1408.318+0.151(R_*t*_)-4.368(T_*t*_)-12.798(H_*t*_)

D_*t *_: Number of DF/DHF patients reported in the month *t*

It was observed that the coefficient of determination (R^2^) for this model was low for the non-rainy season but high for the rainy season (Figure 4).

Multiple regression analysis was carried out for the occurrence of DF/DHF cases for the rainy season. The Empirical Relationship-2 (ER-2) between DF/DHF cases and rainfall, temperature and humidity factors at time *t *(T_*t*_, R_*t *_and H_*t*_) was as follows:

ER-2:

D_*t *_= -13.893+1.3444(T_*t*_)-0.276(H_*t*_)+0.377(R_*t*_)

The coefficient of determination (R^2^) was found as 0.62.

According to the development period from egg to human disease, there is a time lag of about one month that leads to DF/DHF cases occurring during 7 – 45 days. The duration of larvae stages to adult is 7 to 12 days and the lifespan of for female mosquito is about 8 to 15 days [17]. Meantime, the virus develops in the mosquito for a period of 8–10 days. By the time a person infected with dengue virus develops fever the infection is widely disseminated to many people. The virus is found in serum or plasma, in circulating blood cells and in selected tissues, especially those of the immune system, for approximately 2–7 days, roughly corresponding to the period of fever [2]. Thus, DF/DHF cases at time *t *(in month i.e. May) depends on others factors at time *t-1 *(one month before *t *i.e. *t-1 *or the month April). In this empirical relation, the regression coefficients represent the independent contributions of each variable to the prediction of the dependent variable.

The Empirical Relationship- 3(ER-3) with a one-month time lag as shown below offered a coefficient of determination (R^2^) as 0.81. This has shown considerable 31 percent increase than ER-2 and 88 percent than ER-1. The results have shown considerable improvement.

ER-3:

D_*t *_= 621.824+0.345(R_*t*-1_)- 0.609(T_*t*-1_)-6.321(H_*t*-1_)

Where,

*t *= Time (in the unit of month) used as suffix to indicate the month for which the data belong

*t-1 *= one month before the month *t*

**Figure 5:**
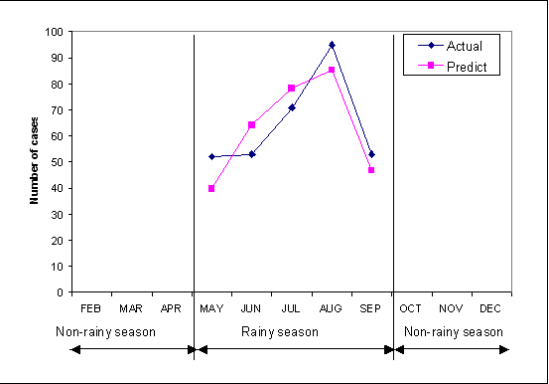
The relationship between actual and forecasted DF/DHF cases in Sukhothai, Thailand in 1998 during rainy season using ER-3.

Therefore, the ER-3 was selected to model the DF/DHF incidence in Sukhothai in as the closest output to the actual data during rainy season and the results were validated with 1998 data (Figure 5).

### Information value computation and analysis

**Figure 6:**
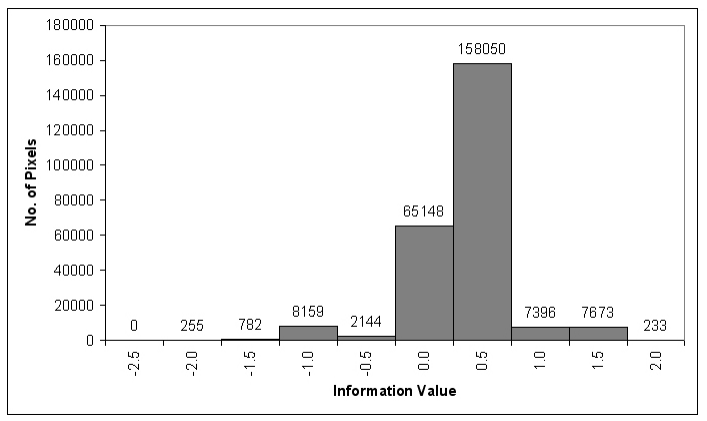
Histogram between information values and area pixels affected by dengue.

Information values were calculated for each land cover type. Negative values indicate low-risk level and the positive values indicate high-risk level of Dengue. Six possible risk classes were identified; Information-value scores obtained from the quartile of a data set at each range of 0.5 were used as the cut-off levels. Risk classes were designated as very low, low, moderately low, moderately high, high and very high respectively with scores as: less than -1.0, -1.0 to -0.5, -0.5 to 0.0, 0.0 to 0.5, 0.5 to 1.0 and greater than 1.0 respectively (Figure 6).

**Figure 7:**
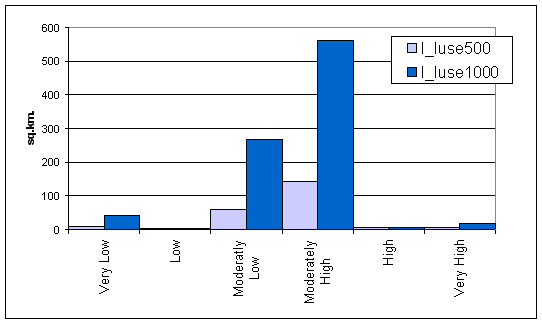
Dengue risk classes using 500 and 1000 m buffer zones.

The highest information value was obtained for the Built-up area. This indicated that Built-up area has the maximum influence on the incidence of dengue. The other classes showing negative values indicate lesser influence on dengue epidemics. Two buffer regions of 500 m and 1000 m in the land use/ land cover maps were analysed for the information value (Figure 7). It was done to investigate if the changes of neighbourhood sizes have any effect on the information value or not.

**Figure 8:**
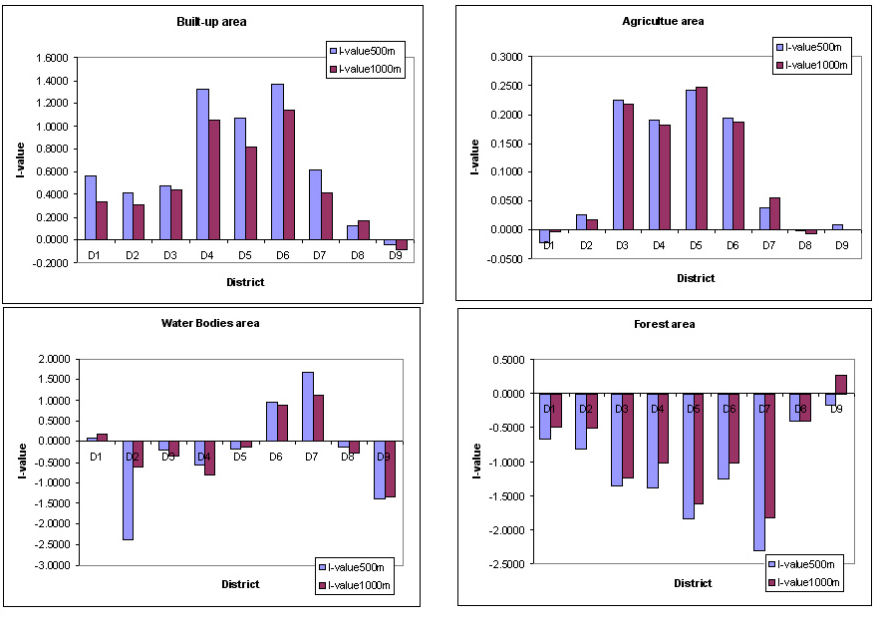
Dengue risk zones received for physical environment using information value.

Information values were clustered in 4 groups: first group represented the high positive values i.e. Built-up (BU) area. Information value in this group represented the highest value that meant built-up category is the highest spatial risk factor in all the districts both for 500 and 1000 m buffer zones. Second group represented the positive relationship with Water Bodies (WB). Information value in this group represented the high values for both buffer regions of 500 and 1000 m in D1, D6 and D7 districts. Third group represented the positive values for the Agriculture areas. Information value in this group represented positive values for both 500 and 1000 m buffer zones that mean the Agriculture category has shown positive influence as a risk factors in D2, D3, D4, D5, D6 and D7 district. Fourth group represented that for all the districts forest (FR) area indicated no risk for dengue, as information values were negative for both 500 and 1000 m buffer zones. The information value obtained using Land use/Land cover risk zone is shown in Figure 8 and Table 3.

**Table 3:** Information value computation and analysis for Sukhothai province in physical factors

Physical Factors	District Code	District Name	I-value 500 m	Risk Classes500	I-value 1000 m	Risk Classes1000
Built-up Area (BU)	D1	Muang	0.56	High	0.33	Moderately High
	D2	Kong Krai Lat	0.41	Moderately High	0.31	Moderately High
	D3	Khili Mat	0.48	Moderately High	0.45	Moderately High
	D4	Thung Saliam	1.32	Very High	1.05	Very High
	D5	Ban Dan Lan Hoi	1.07	Very High	0.82	High
	D6	Si Satchanalai	1.37	Very High	1.14	Very High
	D7	Si Samrong	0.62	High	0.41	Moderately High
	D8	Sawan Khalok	0.12	Moderately High	0.18	Moderately High
	D9	Si Nakhon	-0.04	Moderately Low	-0.08	Moderately Low

Water Bodies (WB)	D1	Muang	0.08	Moderately High	0.17	Moderately High
	D2	Kong Krai Lat	-2.40	Very Low	-0.64	Low
	D3	Khili Mat	-0.20	Moderately Low	-0.34	Moderately Low
	D4	Thung Saliam	-0.56	Low	-0.82	Low
	D5	Ban Dan Lan Hoi	-0.19	Moderately Low	-0.14	Moderately Low
	D6	Si Satchanalai	0.95	High	0.87	High
	D7	Si Samrong	1.66	Very High	1.13	Very High
	D8	Sawan Khalok	-0.13	Moderately Low	-0.28	Moderately Low
	D9	Si Nakhon	-1.41	Very Low	-1.33	Very Low

Agriculture Area (AGR)	D1	Muang	-0.02	Moderately Low	0.00	Moderately Low
	D2	Kong Krai Lat	0.03	Moderately High	0.02	Moderately High
	D3	Khili Mat	0.23	Moderately High	0.22	Moderately High
	D4	Thung Saliam	0.19	Moderately High	0.18	Moderately High
	D5	Ban Dan Lan Hoi	0.24	Moderately High	0.25	Moderately High
	D6	Si Satchanalai	0.19	Moderately High	0.19	Moderately High
	D7	Si Samrong	0.04	Moderately High	0.05	Moderately High
	D8	Sawan Khalok	0.00	Moderately Low	-0.01	Moderately Low
	D9	Si Nakhon	0.01	Moderately High	0.00	Moderately Low

Forest Area (FR)	D1	Muang	-0.67	Low	-0.48	Moderately Low
	D2	Kong Krai Lat	-0.81	Low	-0.51	Low
	D3	Khili Mat	-1.35	Very Low	-1.24	Very Low
	D4	Thung Saliam	-1.38	Very Low	-1.01	Very Low
	D5	Ban Dan Lan Hoi	-1.83	Very Low	-1.61	Very Low
	D6	Si Satchanalai	-1.25	Very Low	-1.00	Very Low
	D7	Si Samrong	-2.30	Very Low	-1.82	Very Low
	D8	Sawan Khalok	-0.40	Moderately Low	-0.39	Moderately Low
	D9	Si Nakhon	-0.16	Moderately Low	0.27	Moderately High

**Figure 9:**
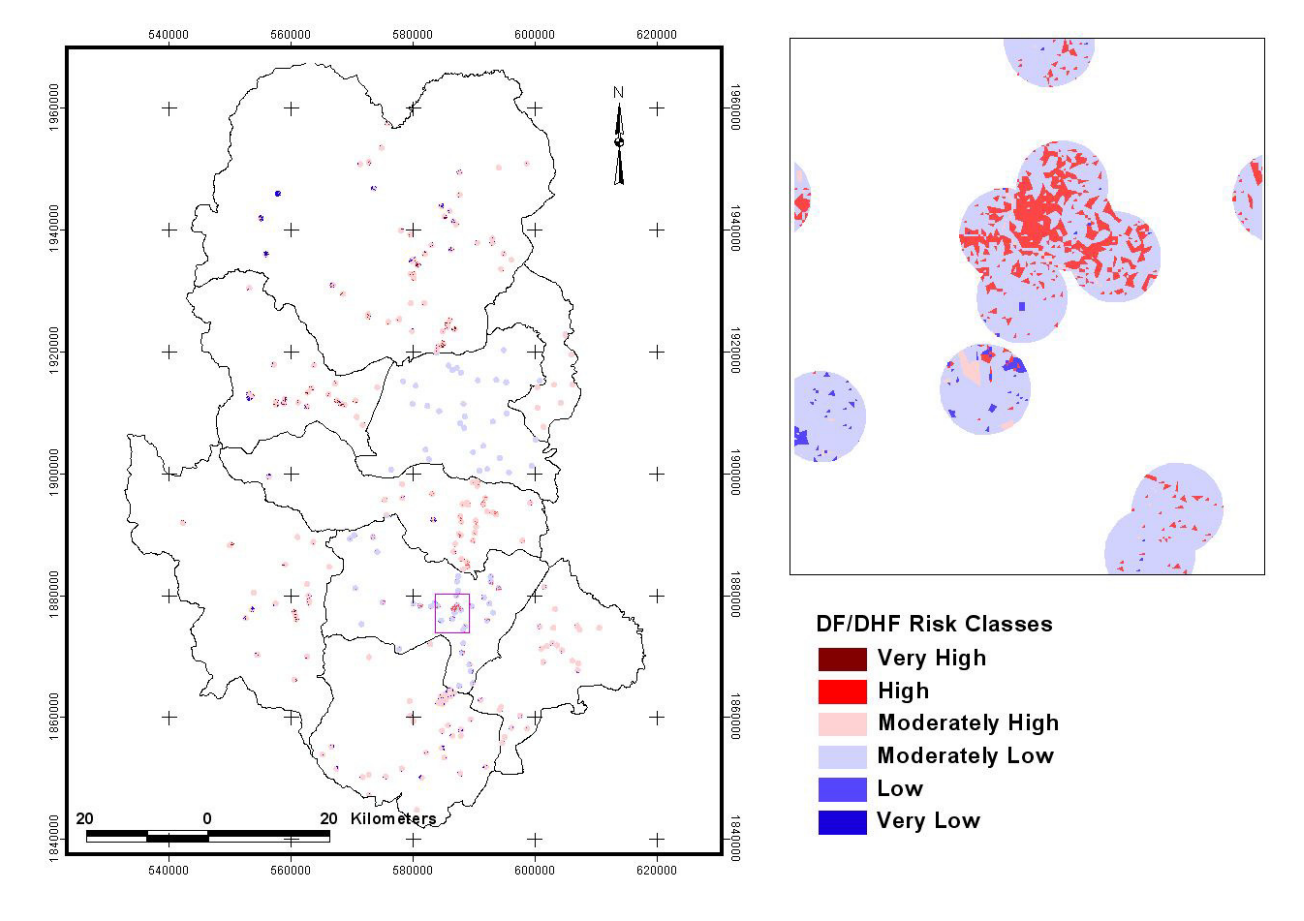
DF/DHF risk zone map of Sukhothai, Thailand.

Most of the risk zones areas are located in the moderately risk class both for 500 meter and 1000 m buffer zones. Information values representing the very high and high-risk classes were in built-up area and water body categories as shown in the Figure 8. Risk zones showing the negative or low risk classes are mostly in the forest area. Sithiprasasna and Linthicum have also shown that DHF incidence was poorly correlated with the forest cover in Tak province of Thailand [24]. It confirms that the DF/DHF cases mostly occur in urban and suburban areas. Table 2 shows the physical categories and their Information Values for each of the districts. Very High Risk zone (information value > 1.0) was in D4, D5, and D6 districts. High Risk zone (information values between 1.0 and 0.5) were observed in D7 and D1 districts. The spatial statistical relationship of various land use/land cover classes with dengue-affected areas was quantified in the form of information value and a dengue risk map was generated as shown in the Figure 9.

## Conclusion

This paper has offered some useful information related to the dengue incidence. Analysis of the climatic factors such as rainfall, temperature and humidity with the dengue incidences has revealed that dengue generally occurred when average temperature rose above normal as found during the El-nino in 1998. It also occurred when the rainfall was comparatively lower and humidity was higher than average.

A composite analysis of these three factors with dengue incidences was carried out using multivariate regression analysis. Three empirical models ER-1, ER-2 and ER-3 were evaluated. It was found that these three factors shave significance and can be related to the find expected number of dengue cases. The results have shown significant high coefficient of determination if applied only for the rainy season using empirical relation-2 (ER-2). These results have shown further improvement once a concept of time lag of one month was applied using the ER-3 empirical relation. ER-3 model is most suitable for the Sukhothai province in predicting possible dengue incidence with 0.81 coefficient of determination.

An analysis of physio-environmental factors such as land use/ land cover types with dengue incidence was carried out. The aim of this analysis was not only to find the effect of physio-environmental factors on dengue incidences but also to find influence of these factors in quantitative terms. It was found that built-up areas have highest influence and constitute the highest risk zones. The agriculture areas offered the second level of high-risk influence. Water bodies posed significant risk in only one district. Forest areas almost do not have any influence on the dengue risk zonation. Information value approach applied to develop the dengue risk map of the Sukhothai province. Dengue risk map can be used by the Public Health Department for applying preventive measures to control the dengue outbreak. This map takes into account the physio-environmental factors and also the mobility and the life duration of the Aedes Aegypti mosquitoes. Public Health Department can initiate their effort once the ER-3 predicts a possibility of significant high dengue incidence. This will help in focussing the preventive measures being applied on priority in very high and high-risk zones and save time and money.

## Authors' contributions

Authors KN and NKT collaborated intensely on all aspects of the manuscript, from research design to data preparation. KN carried out most of the statistical and GIS analysis using Information Value approach and drafted the manuscript. Both authors read and approved the final manuscript. The manuscript was vastly revised and improved based on the reviewers' comments.
